# Complete mitochondrial genome of the litchi pathogen *Phytophthora litchii*

**DOI:** 10.1128/mra.01235-25

**Published:** 2025-12-29

**Authors:** Jinhua Sun, Min Li, Gengxin Chen, Yajun Ran, Xianheng Lv, Meijiao Hu

**Affiliations:** 1 Key Laboratory of Integrated Pest Management on Tropical Crops, Environment and Plant Protection Institute, Chinese Academy of Tropical Agricultural Sciences, Ministry of Agriculture and Rural Affairs 117453, Haikou, China; 2 State Key Laboratory of Green Pesticide, Center for R&D of Fine Chemicals of Guizhou University 71206, Guiyang, China; 3 Guizhou Key Laboratory of Advanced Computing, Guizhou Normal University 12686, Guiyang, China; Rochester Institute of Technology, Rochester, New York, USA

**Keywords:** *Phytophthora litchii*, mitochondrial genome, litchi

## Abstract

The complete mitochondrial genome of the *Phytophthora litchii* strain hk-1 isolated from China was assembled and annotated. The circular genome has a length of 38,126 bp and is AT-rich (78.26%). It harbors two ribosomal RNA genes, 25 transfer RNA genes, and 41 protein-coding genes.

## ANNOUNCEMENT

Litchi (*Litchi chinensis*) is a tropical and subtropical fruit tree that suffers from serious downy blight, caused by *Phytophthora litchii,* formerly *Peronophythora litchii* (1, 2). *P. litchii* is an obligate pathogen of litchi and has been reported to cause over 60% loss in Southern China (3). The *P. litchii* strain hk-1 was isolated from Hainan Province, China (19.899363°N, 110.255165°E). The first genome assembly of *P. litchii* strain SHS3 was generated using Illumina short-read sequencing technology (4), followed by the assembly of its 37,950 bp mitogenome (NC_063792.1) (5). To conduct a comparative analysis of mitochondrial haplotypes, the mitogenome of hk-1 was assembled and annotated.

The hk-1 was cultured in 10% liquid V8 medium at 25°C with shaking at 80 rpm for 1 week. The mycelia were harvested, ground into a powder with liquid nitrogen, and total DNA was extracted using CTAB methods (6). DNA purity was determined using NanoDrop One UV-Vis spectrophotometer (Thermo Fisher Scientific). DNA concentration was quantified with a Qubit 3.0 Fluorometer (Invitrogen, USA). For PacBio HiFi sequencing, SMRTbell 15 kb libraries were constructed following the standard protocol (Pacific Biosciences, CA). Briefly, (i) shearing 8 μg gDNA by g-TUBEs (Covaris, MA); (2) repairing DNA; (3) ligating barcoded hairpin adapters from the SMRTbell Express Template Prep Kit 2.0 (Pacific Biosciences); (iv) nuclease treatment with the SMRTbell Enzyme Clean Up Kit; (v) screening DNA fragments with the BluePippin (Sage Science), and binding to polymerase; (vi) purifying the SMRTbell library using AMPure PB Beads; and (vii) analyzing the library fragments with Agilent 2100 Bioanalyzer (Agilent Technologies, USA). Sequencing was carried out on a PacBio Sequel II platform using Sequencing Primer V5 and Sequel II Binding Kit 2.2. This generated 6,383 CCS reads with >99.0% accuracy, totaling 121.8 Mb of sequencing data with an *N*_50_ of 19.5 kb. From these reads, 1,134 high-depth k-mer reads were selected using BBNorm v39.09 (7) with mindepth = 50 and assembled by Flye v2.9.6 (8) with parameter (--plasmids --pacbio-hifi). Two circular contigs were assembled: one is the complete mitochondrial genome, and the other is rDNA repeated unit. The mitogenome was annotated using ORFfinder and GeSeq (9), with that of SHS3 (NC_063792.1) as RefSeq. The start and stop positions of coding genes were manually verified using IGV (10).

**Fig 1:**
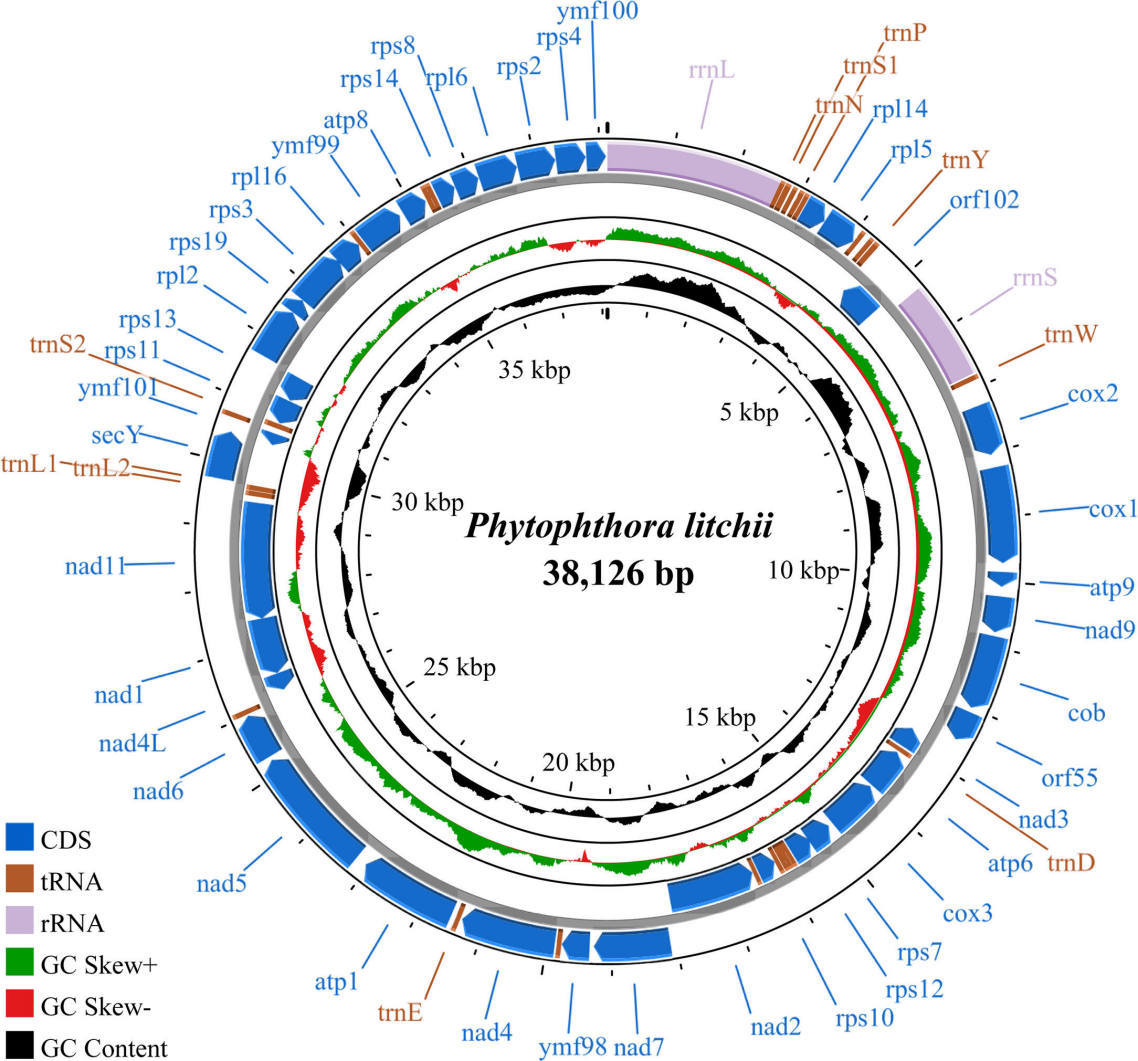
The mitochondrial genome map of *P. litchii*. The outermost circle illustrates gene arrangement, with arrows indicating transcriptional orientation. The second circle indicates GC content, the third circle shows the GC skew, and the innermost circle shows sequence length. Color coding distinguishes different gene categories, as detailed in the legend at the bottom left.

The mitogenome is 38,126 bp in length, with an AT content of 78,3%. Visualization using the Proksee server (11), which illustrates the transcription directions, gene locations, and GC content (Fig. 1). The mitogenome contains 68 genes, including 41 protein-coding genes (PCGs), 25 transfer RNA (tRNA), and two ribosomal RNA (rRNA) (Table 1). All the PCGs initiate with ATG and terminate with TAA, except for nad11, which terminates with TGA. The mitogenome of hk-1 covers the entire that of SHS3 at 96.6% identity, with 1,068 SNPs and 52 InDels. Additionally, two novel ORFs (orf55 and orf102), with 156 and 209 amino acids, respectively, were identified and validated by blastp against the ClusteredNR database. This work presents the complete mitochondrial genome sequence of *P. litchii* strain hk-1, providing valuable data to genetic and evolutionary studies of this species.

**TABLE 1:** The gene content, organization, and codon usage in the mitochondrial genome of *P. litchii* strain hk-1

Gene	Type	Location	Size (bp)	Direction	Intergenic region length (bp)	Start/stop codons
rrnL	rRNA	1–2,653	2,653	Positive	6	
trnN	tRNA	2,660–2,731	72	Positive	12	
trnS1	tRNA	2,744–2,832	89	Positive	32	
trnM_0	tRNA	2,865–2,936	72	Positive	40	
trnP	tRNA	2,977–3,051	75	Positive	13	
trnM_1	tRNA	3,065–3,137	73	Positive	14	
rpl14	CDS	3,152–3,523	372	Positive	6	ATG/TAA
rpl5	CDS	3,530–4,063	534	Positive	7	ATG/TAA
trnG_1	tRNA	4,071–4,142	72	Positive	89	
trnG_0	tRNA	4,232–4,303	72	Positive	21	
trnY	tRNA	4,325–4,408	84	Positive	48	
orf102	CDS	4,457–5,086	630	Negative	187	ATG/TAA
rrnS	rRNA	5,274–6,780	1,507	Positive	29	
trnW	tRNA	6,810–6,881	72	Positive	375	
cox2	CDS	7,257–8,033	777	Positive	199	ATG/TAA
cox1	CDS	8,233–9,711	1,479	Positive	135	ATG/TAA
atp9	CDS	9,847–10,074	228	Positive	150	ATG/TAA
nad9	CDS	10,225-10,782	558	Positive	58	ATG/TAA
cob	CDS	10,841–11,995	1,155	Positive	70	ATG/TAA
orf55	CDS	12,066–12,536	471	Positive	183	ATG/TAA
nad3	CDS	12,720–13,073	354	Negative	35	ATG/TAA
trnD	tRNA	13,109–13,182	74	Negative	25	
atp6	CDS	13,208–13,927	720	Negative	29	ATG/TAA
cox3	CDS	13,957–14,871	915	Negative	75	ATG/TAA
rps7	CDS	14,947–15,378	432	Negative	−26	ATG/TAA
rps12	CDS	15,353–15,733	381	Negative	22	ATG/TAA
trnV	tRNA	15,756–15,828	73	Negative	2	
trnI	tRNA	15,831–15,904	74	Negative	2	
trnQ	tRNA	15,907–15,978	72	Negative	14	
trnR_0	tRNA	15,993–16,066	74	Negative	4	
rps10	CDS	16,071–16,397	327	Negative	18	ATG/TAA
trnF	tRNA	16,416–16,489	74	Negative	6	
nad2	CDS	16,496–17,989	1,494	Negative	111	ATG/TAA
nad7	CDS	18,101–19,279	1,179	Positive	63	ATG/TAA
ymf98	CDS	19,343–19,771	429	Positive	4	ATG/TAA
trnH	tRNA	19,776–19,847	72	Positive	30	
nad4	CDS	19,878–21,353	1,476	Positive	24	ATG/TAA
trnE	tRNA	21,378–21,449	72	Positive	79	
atp1	CDS	21,529–23,058	1,530	Positive	173	ATG/TAA
nad5	CDS	23,232–25,226	1,995	Positive	60	ATG/TAA
nad6	CDS	25,287–25,997	711	Positive	21	ATG/TAA
trnR_1	tRNA	26,019–26,091	73	Positive	59	
nad4L	CDS	26,151–26,453	303	Negative	2	ATG/TAA
nad1	CDS	26,456–27,436	981	Negative	−4	ATG/TAA
nad11	CDS	27,433–29,433	2,001	Negative	102	ATG/TGA
trnL1	tRNA	29,536–29,618	83	Negative	10	
trnL2	tRNA	29,629–29,712	84	Negative	11	
secY	CDS	29,724–30,467	744	Positive	−9	ATG/TAA
ymf101	CDS	30,459–30,662	204	Negative	20	ATG/TAA
trnC	tRNA	30,683–30,753	71	Positive	3	
trnS2	tRNA	30,757–30,841	85	Negative	13	
rps11	CDS	30,855–31,274	420	Negative	12	ATG/TAA
rps13	CDS	31,287–31,703	417	Negative	28	ATG/TAA
rpl2	CDS	31,732–32,535	804	Positive	3	ATG/TAA
rps19	CDS	32,539–32,775	237	Positive	3	ATG/TAA
rps3	CDS	32,779–33,609	831	Positive	−23	ATG/TAA
rpl16	CDS	33,587–33,991	405	Positive	2	ATG/TAA
trnM_2	tRNA	33,994–34,067	74	Positive	20	
ymf99	CDS	34,088–34,765	678	Positive	61	ATG/TAA
atp8	CDS	34,827–35,219	393	Positive	21	ATG/TAA
trnK	tRNA	35,241–35,313	73	Positive	3	
trnA	tRNA	35,317–35,389	73	Positive	25	
rps14	CDS	35,415–35,714	300	Positive	8	ATG/TAA
rps8	CDS	35,723–36,103	381	Positive	11	ATG/TAA
rpl6	CDS	36,115–36,720	606	Positive	9	ATG/TAA
rps2	CDS	36,730–37,320	591	Positive	8	ATG/TAA
rps4	CDS	37,329–37,790	462	Positive	8	ATG/TAA
ymf100	CDS	37,799–38,101	303	Positive	24	ATG/TAA

## Data Availability

The mitochondrial genome of *P. litchii* is available in ENA under accession number OZ349077.1. The associated BioProject, BioSample, and SRA numbers are PRJEB101184, SAMEA120409876, and ERR15751565, respectively.
